# Evolution of Nanoparticle-Mediated Photodynamic Therapy: From Superficial to Deep-Seated Cancers

**DOI:** 10.3390/molecules24030520

**Published:** 2019-01-31

**Authors:** Maharajan Sivasubramanian, Yao Chen Chuang, Leu-Wei Lo

**Affiliations:** Institute of Biomedical Engineering and Nanomedicine, National Health Research Institutes, Zhunan 350, Taiwan; siva.mdu@gmail.com (M.S.); ycchuang@nhri.org.tw (Y.C.C.)

**Keywords:** single-photon, two-photon, X-ray, photosensitizer, photodynamic therapy, PDT

## Abstract

Enthusiasm for photodynamic therapy (PDT) as a potential therapeutic intervention for cancer has increased exponentially in recent decades. Photodynamic therapy constitutes a clinically approved, minimally invasive treatment modality that uses a photosensitizer (light absorbing molecule) and light to kill cancer cells. The principle of PDT is, when irradiated with a light of a suitable wavelength, a photosensitizer absorbs the light energy and generates cytotoxic free radicals through various mechanisms. The overall efficiency of PDT depends on characteristics of activation light and in-situ dosimetry, including the choice of photosensitizer molecule, wavelength of the light, and tumor location and microenvironment, for instance, the use of two-photon laser or an X-ray irradiator as the light source increases tissue-penetration depth, enabling it to achieve deep PDT. In this mini-review, we discuss the various designs and strategies for single, two-photon, and X-ray-mediated PDT for improved clinical outcomes.

## 1. Introduction

Chemotherapy and radiotherapy constitute the two major clinical treatment modalities for cancer, which often cause deleterious side effects resulting in poor clinical outcomes [1,2,3,4]. As an alternative, photodynamic therapy (PDT) is emerging as a potential therapeutic regime due to its highly effective, non-invasive, localized therapy with minimal or no damage to healthy tissues and a superior healing process [5,6,7,8,9]. Integral to PDT are a photosensitizer (PS), a light-absorbing molecule, and a light source with a suitable wavelength [10]. When irradiated, PS absorbs the light energy and makes a transition to an excited state. The excited PS then undergoes a photochemical reaction (PR) with a biological environment in the cancer cells to generate cytotoxic reactive oxygen species (ROS), and this overall process is called PDT. There are two main types of PDT, type I PDT involves electron transfer PR to generate radical and radical anion species, whereas type II PDT directing PR via energy transfer between oxygen and excited PS to produce singlet oxygen—a highly reactive and cytotoxic ROS [11,12,13,14,15]. Abundant ROS produced during the PDT are responsible for cancer-cell death through co-operative effects of the immune system and by apoptosis or necrosis [16,17].

Although the promise generated by PDT is far-reaching, it suffers from certain limitations, which are due to the inherent properties of small molecules PS, e.g., (1) most PSs have poor solubility in aqueous solution and easily aggregate after administration due to their π–π stacking and hydrophobic interaction that makes it very difficult to formulate them adequately and sometimes extremely decreases their photodynamic activity against tumors; (2) poor selectivity between diseased and healthy cells, and (3) limitation of PS delivery. In addition, a number of factors in relation to its therapeutic efficacy are necessary to consider, such as initial oxygen concentration in tumor microenvironment, penetration depth of the light, the light intensity and wavelength utilized, and their complemented PS. For these reasons, the performances of clinical PDT to date have been far from optimal, and current PDT is mainly focused on superficial cancers, including skin, retina, bladder, esophageal, lung, gastrointestinal tract, and head and neck cancers [18].

Recently, nanomaterials have been used in different aspects of cancer management. More specifically, nanotechnology is attractive in PDT for several reasons [19,20,21,22]: (1) In nanoparticle (NP)-based PS delivery systems, the high surface-to-volume ratio results in high PS loading capability; (2) enhanced PS concentration at the desired site and reduced transition into normal tissues is achieved either by attaching ligands that include tumor-specific antibodies or proteins (active transport) [23,24,25] or through an enhanced permeability and retention (EPR) effect [26,27] (passive transport), avoiding undesirable non-specific distributions; (3) their ability to accommodate PS as guest molecules, which enhance their water solubility and biocompatibility; (4) the excitation properties of the PS are well preserved when encapsulated in the NP, resulting in large extinction coefficients and improved quantum yields; (5) NPs, especially inorganic NPs, have unique size-tunable optical properties that can match the working region of PS; (6) impart multifunctional features, such as simultaneous diagnostic imaging and therapy (theranostics). As a result, NP-based PS delivery systems consist of various types of organic and inorganic compounds that have been studied and listed in Table 1, and which demonstrate that the development of NP-mediated PDT is highly beneficial.

**Figure 1:**
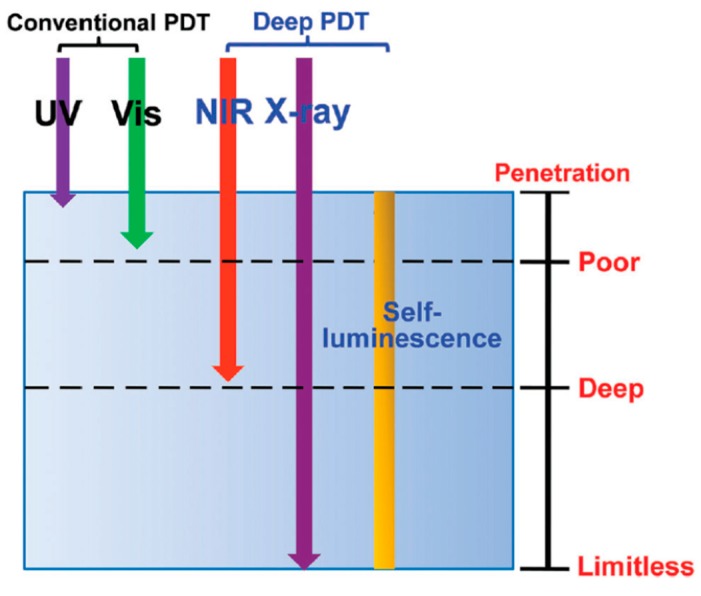
Comparison of tissue penetration of different excitation sources. Reprinted with permission from reference [35]. Copyright 2016 Royal Society of Chemistry.

Successful PDT depends on the choice of PS, appropriate excitation light, and tumor type. Note that biological components have less absorption in the region between 600 and 1200 nm; light at this region can deeply penetrate biological tissue. Otherwise most PSs are activated at 600–700 nm [28,29,30]. The conventional PDT uses PS, which is activated by this activation wavelength and suffers from poor penetration depth, and thus its application is limited to superficial tumors. In recent years, apart from the above-discussed NP-based PS platform, deeper PDT uses two-photon excitation [31], X-ray [32], or self-luminescence [33] as the light source [34] to provide better penetration ability to treat tumors lying under deep tissues (Figure 1). The difference between single-photon excitation and two-photon excitation (TPE) is that, in TPE specifically, absorption increases with the square of light intensity, allowing three-dimensional selectivity to target tumor cells. Besides two-photon, X-rays and self-luminescence with limitless penetration depth in the human body are also excellent light sources of deep PDT.

In this review, we highlight the design, methodology, and promising research contributions of single-photon, two-photon, and X-ray PDT. We hope that this review will provide crucial ideas for the creation of precise PDT platforms for successful clinical translation.

## 2. Single-Photon PDT

Commonly used PS for single-photon PDT, such as derivatives of porphyrin, phthalocyanine, chlorin (Ce6), etc., are activated by excitation light in the wavelength range of ~600–690 nm. Because biological components have less absorption in the region between 600 and 1200 nm, light at this region can deeply penetrate biological tissue. This section intends to give a few examples of single-photon PDT, as summarized below.

Lo et al. developed nano PS based on mesoporous silica (MS) NP and Pd-porphyrins (PdTPP) for cancer cell based PDT [36]. The well-ordered nano-channels of MS allowed successful conjugation and facilitated the uptake of PdTPP in breast cancer cells. When irradiated with 532 nm light, the composite nanoplatform demonstrated photo-induced cytotoxicity by increasing the intracellular density of ROS. In another work, the same research group developed a theranostic PDT (MS-PdTPP) system, which consists of PdTPP as a PS, NIR dye ATTO 647N for optical imaging, and cyclic RGD peptides for targeting αvβ_3_ overexpressing cancer cells [37]. In vitro, the nanoplatform was selectively taken up by U87MG cell and exhibited an excellent therapeutic effect upon irradiation with 532 nm light. The authors suggest that the multifunctional nanoplatform developed in this study could constitute a useful PDT theranostic.

Lee et al. developed calcium carbonate (CaCO_3_)-mineralized polymeric NP as a potential ultrasound (US)-guided PDT of tumors [38]. The PDT system consists of Ce6-loaded CaCO_3_-mineralized PEG-*b*-PAsp copolymer. In the presence of tumoral acidic pH (6.4), Ce6 was rapidly released from the copolymer with the formation of CO_2_ bubbles, which is due to the decomposition of CaCO_3_. In MCF-7 breast cancer cells, mineralized NP demonstrated photo-induced cytotoxicity with increasing Ce6 concentration compared to free Ce6. The mineralized PDT nanoplatform could be considered as a useful theranostic system for US imaging-guided PDT.

One of the main challenges faced by PDT in cancer is tumor hypoxia, which is deleterious to PS that requires oxygen for the generation of cytotoxic ROS. Hypoxia is a condition characterized by an insufficient oxygen supply, which is a prominent feature of solid tumors. Hypoxia significantly reduces the therapeutic outcome of PDT and poses a major threat to clinical translation [39,40,41,42,43]. To solve this problem, oxygen-evolving PDT systems are conceived as a potential alternative. 

**Figure 2:**
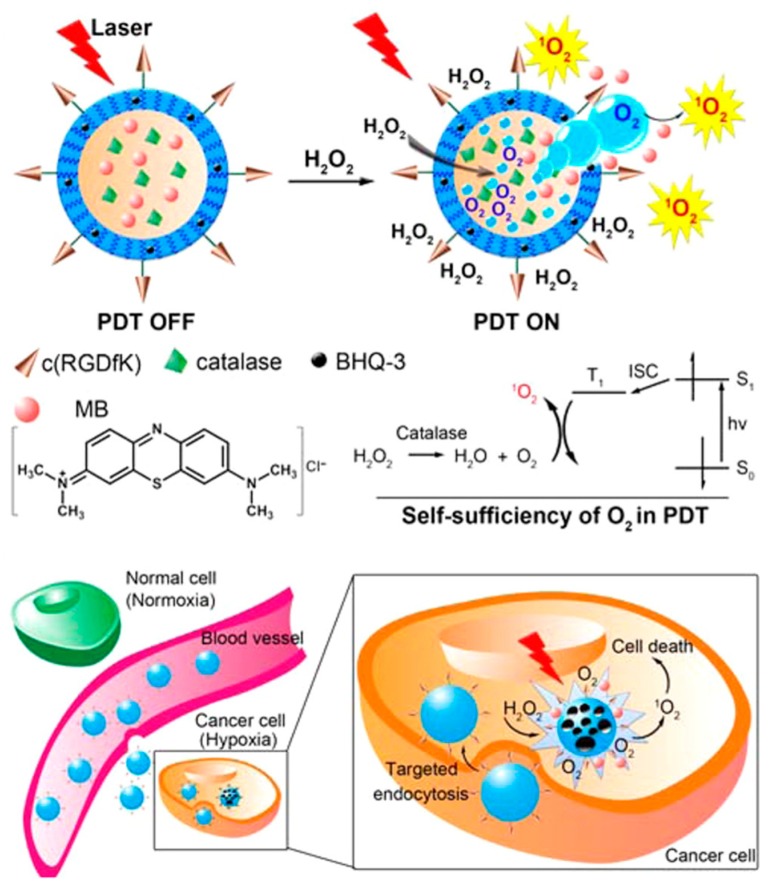
Schematic illustration of self-sufficiency of O_2_ in photodynamic therapy (PDT). Reprinted with permission from reference [44]. Copyright 2015 American Chemical Society.

Guo et al. developed an O_2_-evolving highly selective PDT nanoplatform for hypoxic tumors. The PDT system is a poly(d,l-lactic-co-glycolic acid) (PLGA) NP loaded with methylene blue (MB) as PS and enzyme catalase (CS) as an O_2_-evolving agent in the aqueous core, a black hole quencher (BHQ) in the polymer shell, and finally conjugated with cyclic RGD peptide for targeting αvβ_3_-overexpressing cancer cells [44]. In the presence of U87MG cells, intracellular H_2_O_2_ permeates the NP and generates O_2_ (which is essential for ROS production under irradiation) through catalase activity (Figure 2). The release of PS by NP shell erosion promotes localized PDT. After intravenous administration of the nanoplatform in glioma tumor-bearing mice, irradiation with 635 nm light resulted in complete tumor elimination after 7 days of treatment.

Recently, Hyeon et al. developed a theranostic PDT nanoplatform against hypoxic tumors [45]. The PDT nanoplatform consists of Ce6 loaded manganese ferrite (MnFe_2_O_4_) anchored MS NP (MFMSN), in which MnFe_2_O_4_ plays a dual role as O_2_-evolving agent and as T_2_-contrast agent for in vivo magnetic resonance imaging (MRI). Catalytic activity of MFMSN supplied O_2_ continuously, thereby reducing hypoxia levels in in vitro and in vivo models. In vivo studies in a tumor model showed preferential accumulation of Ce6-loaded MFMSN at tumor due to EPR-effect evidence by MRI and exhibited excellent PDT effect by attenuating hypoxia.

## 3. Two-Photon PDT

Traditional PS have a strong absorption band around 400 nm (Soret band) and a satellite absorption band between 600–800 (Q band), which limits tissue penetration ability and thus results in poor PDT. Despite the above-mentioned advantage of upconversion NP for PDT, the low quantum yield of upconversion luminescence of upconversion NP (less than 1% for most of UCNPs) has been an important issue for limiting the use of PDT [46]. As an alternative, TP-excited PS was developed. PS with a TP cross-section can absorb two low energy photons simultaneously and emit higher energy photons. In this process, photon absorption increases with the square of the light intensity, allowing spatially selective PDT. Because the two-photon PS can absorb NIR light, deep tissue penetration can be achieved, resulting in enhanced PDT. In this section, we summarize various NP-based two-photon PDTs [35,47,48]. 

**Figure 3:**
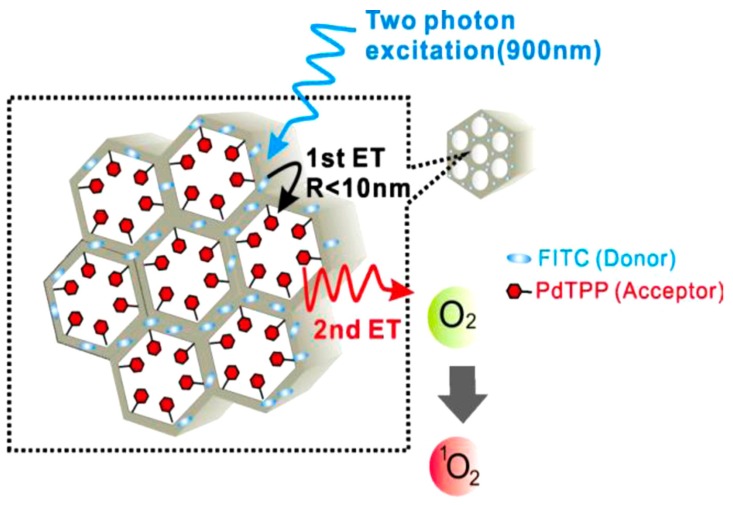
Two-step intra-MS energy transfer from FITC (framework) to PdTPP (nanochannels) to generate reactive oxygen species (ROS) by two-photon excitation (TPE). Reprinted with permission from reference [50]. Copyright 2011 Elsevier.

Recently, Gary-Bobo et al. covalently encapsulated porphyrin derivative as TP PS in mannose-functionalized MS NP [49]. The covalent conjugation with MS NP retained TPA properties of the PS, leading to enormous TPA cross-sections (up to 8 Mega-Goeppert−Mayer units) for a single MS NP, and exhibited strong photo-induced cytotoxicity upon irradiation with a 760 nm laser. When systemically administered in nude mice bearing HCT-116 xenografts, mannose-MS NP accumulated in the tumor and demonstrated strong PDT effects on irradiation with a 760 nm laser, confirmed by a significant reduction in tumor volume compared to control groups. Lo et al. co-encapsulated fluorescein isothiocyanate as TP absorption antenna and PdTPP as PS in different topological domains of MS NP, to generate an intraparticle energy transfer relay system [50]. The well-ordered mesoporous structure of MS NP facilitated the controlled energy transfer rate up to an unprecedented 93%. Then, photo-induced cytotoxicity following the energy transfer was demonstrated in both in vitro and in vivo breast cancer models (Figure 3).

Velusamy et al. synthesized a new TPA PS molecule 11,12-dibutoxydibenzo[*a,c*]-phenazine bridged amine, and successfully encapsulated it in porous silicon NP to improve biocompatibility and water solubility [51]. The encapsulated PS molecules have considerably high TPA cross-section (7000 GM excited at 800 nm). In vitro, these composite NP induced a significant 40% mortality in macrophage cells upon 800 nm fs pulsed laser irradiation. Kim et al. co-encapsulated aggregation-enhanced two-photon absorbing fluorescent dye aggregates of 9,10-bis(4′-(4″-amino-styryl)styryl)anthracene (BDSA) as a donor and PS 2-devinyl-2-(1-hexyloxyethyl)pyropheophorbide (HPPH) as an acceptor in organically modified silica NP (ORMOSIL) [52]. After irradiation with two-photon light (850 nm), BDSA efficiently up-converted the energy and transferred it to the co-encapsulated HPPH molecules through the intraparticle FRET, which resulted in enhanced generation of singlet oxygen. In vitro, composite NP induced cytotoxicity after two-photon irradiation compared to control groups.

Among the gold (Au) nanomaterials, Au nanorods (Au NR) have been widely utilized in biomedical applications due to their excellent optical properties, large two-photon cross-section, and ability to absorb NIR light due to their strong surface plasmon resonance [53,54,55,56]. Considering these advantages, Chen et al. developed a two-photon activated PDT system using MS NP coated Au NR (MS-AuNR) encapsulating organic PdTPP as PS [57]. The function of MS NP coating is two-fold: (1) it preserves TP absorption properties of Au NR, and (2) it enables high PS loading. When irradiated with two-photon light, excited Au NR activates PS through intra-particle plasmonic resonance energy transfer, resulting in ROS production. In tumor-bearing mice in vivo, irradiation of MS-AuNR-PdTPP with an 800 nm fs laser exhibited excellent PDT effects. Similarly, Zhao et al. developed a core-shell NP that consists of Au NR as a core and silica shell with covalently binding porphyrin molecules (T790) [58]. A silica shell thickness of 20 nm was determined to be the ideal distance between Au NR and T790 molecules to generate optimum two-photon excitation fluorescence. In vitro studies with HepG2 cells showed that irradiation of composite NP with an 800 nm fs laser for 8 min produced ROS that resulted in 50% cell death. 

## 4. X-ray PDT

Most of these PSs have their major Soret band absorption at approximately 400 nm and present several Q-bands extending as far as the 630 nm region. Generally, the excitation of the PS at the Soret band can be much more efficient than that at the Q-band. However, due to the limited penetration depth of UV/blue light, NIR/red light is commonly used to activate the weak Q-band of PS, which is less effective in activating PS in practical PDT due to the narrow energy gap and relatively rapid non-radiative transition. The two-photon activated PDT might constitute a potential solution for deep tumor treatment. However, for practical applications, NIR light can only penetrate 5 mm and the TP focal section is generally less than 1 mm into the tissue; thus, treatment efficiency may still be highly surface-weighted. In an effort to overcome these challenges, X-ray excited optical luminescence (XEOL) constitutes a promising technology to convert X-ray photons to UV/visible photons [32]. X-ray is one of the oldest and most commonly used modalities, and has been in the clinical workplace for more than half a century. Due to limitless depths achievable and interaction between high-energy photons and high Z substances, various types of NP are employed as scintillators to absorb incoming X-rays and activate nearby PSs to allow a high tissue penetrating depth and excitation of locally-loaded PSs when they are delivered in deep-seated tumors. Recently, several types of nanoparticles have been developed for this purpose, such as metal–(in)organic clusters [59,60,61], metal materials [62,63,64], radioluminescent nanophosphors [65,66], and QDs [67], which are summarized and listed in Table 2.

**Figure 4:**
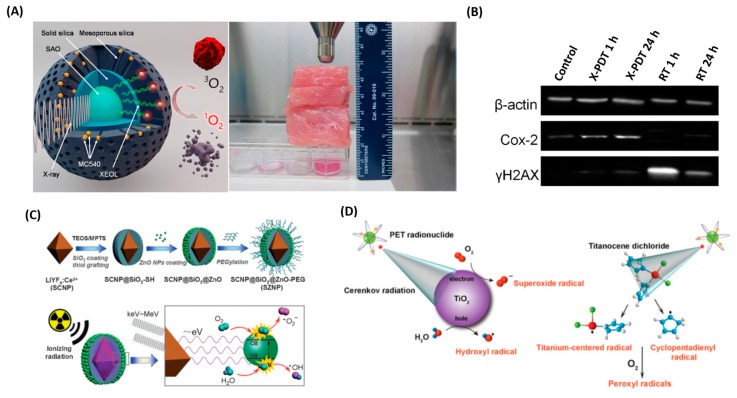
(**A**) Schematic illustration of the working mechanism of X-PDT. Under X-ray irradiation, SAO converts X-rays to visible light photons. The visible light photons, with 4.5 cm thick pork positioned between the X-ray source and cells, activate nearby MC540 molecules to produce cytotoxic ^1^O_2_ that destroys cancer cells in the proximity. Reprinted with permission from references [77]. (**B**) Western blot assays, which further confirm the impact of X-PDT on DNA and membrane lipids. Reprinted with permission from references [78]. (**C**) Schematic illustration of the synthetic route and the mechanism of ionizing radiation-induced PDT. The electron–hole (e^−^–h^+^) pair is formed after exposure to ionizing radiation. Reprinted with permission from references [85]. (**D**) Schematic of CR-mediated excitation of TiO_2_ NP to generate cytotoxic hydroxyl and superoxide radicals from water and dissolved oxygen, respectively. Reprinted with permission from references [33].

A pilot study performed by Chen et al. demonstrated that radiation therapy with PDT could enable the use of lower doses of radiation. Upon exposure to ionizing radiation, such as X-rays, BaFBr:Eu^2+^ displayed luminescence that matched the absorption spectra of PS photofrin perfectly, which, in turn, activated PSs to generate ^1^O_2_ for cancer cell destruction [89]. The authors also demonstrated that radiation doses could be decreased significantly as persistent luminescence NPs were employed. In other words, continuing emissions prolonging the activated time of PSs could generate enough photons for PDT in a short time period. In another study, Zou’s group reported that this strategy was able to induce oxidative stress, mitochondrial damage, and DNA fragmentation on prostate cancer cells in vitro upon a 3 Gy X-ray irradiation [82]. More recently, Xie et al. developed a novel SrAl_2_O_4_:Eu^2+^ (SAO) as X-ray inducible nanoscintillators and demonstrated that tumor growth was efficiently slowed even when 2-cm thick pork muscle tissues were positioned between the X-ray source and the tumor (Figure 4A,B) [77,78]. This is in stark contrast to conventional PDT. X-ray PDT technology is essentially a RT and PDT combination that not only caused oxidative degradation of unsaturated lipids and surface proteins, and induced short-term cell necrosis, but also caused DNA damage and reduced tumor survival in the long run. The key factors influencing X-ray PDT efficiency include X-ray dose, concentration of molecular oxygen, and efficiency of intersystem crossing. In the same group, a low fluence rate and low dose X-ray were implemented to combat radioresistant tumors. Therefore, the additional oxygen could recruit into the tumor region during the period of treatment and enhance singlet oxygen generation. It is noteworthy that, for the type II pathway, PDT effect is highly dependent on oxygen content to generate cytotoxic singlet oxygen, but unfortunately the inner region of a tumor is commonly hypoxic due to abnormal microcirculation and insufficient blood supply. Busch et al. further demonstrated the associated dilemma that PDT creates a hypoxia microenvironment in tissue adjacent to perfused blood vessels and cannot exhibit sustained tumor regression after exhausting oxygen [90]. It has also been reported that several strategies could recruit the availability of oxygen in the tumor during PDT treatment, including hyperbaric oxygen enrichment [91,92], prevention of vascular shutdown [93,94], modulation of fluence rate [77,95], and in situ oxygen self-supplement [96,97]. On the other hand, the oxygen-independent manner utilizing type I photo-oxidative reaction may address the potential issue of insufficient oxygen. Zhang et al. employed Ce^III^-doped LiYF_4_ as X-ray inducible nanoscintillators. It was found that the emitted numerous photons of low energy could match the bandgap of surface-bound ZnO NP, and generated radical and radical anion species (e.g., O_2_^•^^−^, HO^•^) [85] (Figure 4C). Metal oxide NPs are excellent regenerative photocatalysts and could produce ROS through photoexcited electrons and holes reacting with an aqueous electron acceptor (i.e., O_2_) and donor (i.e., H_2_O and OH^−^) [98,99]. This type I mechanism is an oxygen-independent process that led to increased production of anion radicals and significantly improved PDT efficacy under hypoxic conditions.

Similar to the X-ray induced PDT process, Cerenkov radiation mediated PDT was reported by Achilefu et al. in 2015 [33] (Figure 4D). The radionuclide emitting low-intensity Cerenkov luminescence (CL) was employed as an internal light source to activate radical formation, significantly decreasing the cell viability of PS-loaded tumor cells. Furthermore, according to the requirement, the same group also introduced either ^18^F (with a half-life of 1.83 h) or ^64^Cu (with a half-life of 12.7 h) to deliver rapid or sustained CL for Cerenkov-radiation mediated PDT. This approach of internal CL is attractive due to the potential utilization of PDT with clinical radiotracers already in routine use. Another interesting strategy that has great potential was reported in a preliminary study that combines external beam therapy, in which the total light fluence of CL (nJ cm^−2^) is much less than that from X-ray (mJ cm^−2^) [100,101]. 

## 5. Encapsulation of PS in NP

Due to their hydrophobic nature, many PS molecules aggregate in physiological environments after administration. As a result, PS suffer from poor in vivo circulation times and reduced quantum yield of ROS generation. This problem can be solved by encapsulation with NP, which can be either by physical interactions or covalent conjugation. Here we describe PS encapsulation methods in various NPs; Lo et al. encapsulated PdTPP as PS in the MSN nanochannels by covalent conjugation. Briefly, PdTPP was silane modified with aminopropyl trimethoxy silane, followed by condensation with template extracted MSN at 60 °C for 24 h. Then PS conjugated MS NP was purified by repeated centrifugation and washing with distilled water and ethanol; the loading weight % of PdTPP was calculated to be 4.4 [36]. Based on the electrostatic interactions, Lee et al. successfully encapsulated Ce6 in the PEG-b-PAsp NP by biomineralization. The addition of CaCl_2_ solution to PEG-b-Asp solution resulted in the binding of Ca^2+^ ions to the negatively charged aspartic acid. Then, the added Ce6 also bound to Ca^2+^ ion. Finally, biomineralization was completed by the addition of Na_2_CO_3_. Ce6 loaded NP was purified by dialysis, and the loading efficiency was calculated to be 83.3% [38]. Guo et al. used MB as PS was encapsulated in PLGA NP in the aqueous core along with enzyme CS and BHQ in the hydrophobic PLGA shell. The PLGA NP was prepared by double emulsion method; briefly, primary emulsion was carried out by ultrasonication of the mixture of aqueous polyvinyl alcohol (PVA) solution and PLGA solution in dichloromethane containing the respective loading molecules. The resulting primary emulsion was treated again with PVA solution and emulsified to form water-in-oil-in-water (W-O-W) double emulsion. Finally, the solvent was evaporated and washed with ultrapure water to yield PLGA NP with MB and catalase loading efficiency of 19.5 % and 13.1%, respectively [44].

Bazylińska et al. developed dicephalic anionic surfactants for stabilization of the polyelectrolyte multilayer nanocapsules with both solid and liquid cores. The model photosensitizers were successfully loaded into liquid/solid core nanocapsules by nanoemulsification and nanoprecipitation methods. For instance, in nanoprecipitation method, an organic solution containing PLA and porphyrin dye was added dropwise under stirring to aqueous solution containing a surfactant, and the stirring was continued for 1 h. After the solvent evaporation, the solid cores were covered by polyelectrolytes through layer-by-layer adsorption. Authors suggested that the NP prepared in this study could enhance the photostability and in vivo biodistribution of PS [102]. Besides, the same group developed an upconversion NP and successfully loaded into PLGA-origin nanocarrier by W-O-W double emulsion evaporation method. Authors found that the encapsulation process did not affect the optical properties of upconversion NP and could serve as an efficient fluorescent agent for tracking NP within cells and delivery of therapeutic cargos [103]. In another work, Bazylińska et al. developed cubosomes, a polymer-free cubic bicontinuous liquid crystalline dispersions, as carriers for PS. The carrier was synthesized by the dispersion of an optimum amount of phospholipid, monoolein and propylene glycol in water using an ultrasonic processor. Encapsulation was done by dispersing the PS in the melted precursors by ultrasonic bath before dispersion in water. The NP developed in this study exhibited good biocompatibility and simultaneous bioimaging and PDT effects in vitro [104]. 

## 6. Conclusions

After more than a century since PDT was discovered, it has been clinically applied to various tumors and non-malignant diseases, including infections. However, the limited penetration depth of light restricts traditional PDT to superficial tumors. In the last 20 years, numerous significant breakthroughs have been made in PS design to improve photochemical efficiency in deep locations. Recent advances in nanotechnology then opened up an extremely promising avenue in the field of PDT, overcoming major limitations, such as poor solubility, off-targeting, and short plasma half-life. Furthermore, incorporation of two-photon or X-ray excitation to nanoparticle-platform based PSs enables one to deliver a suitable light source into deep regions and activate PDT. In this review, we briefly introduced a variety of approaches to activate PDT to expand our cancer treatment options. Although there is a relative lack of clinical evidence in PDT, when sufficient knowledge has been accumulated, it is anticipated that it may be possible to utilize PDT as a first-line treatment option. Moreover, since the immune-stimulating effects of PDT are well demonstrated in preclinical models, it is possible that PDT could efficiently destroy primary tumors and evoke the immune system to seek out and destroy distant tumors. Thus, we highly anticipate seeing successful translations of the combinational treatment for deep-seated cancers with precision nanoparticle-mediated PDT and self-stimulated immunotherapy in the near future.

## Figures and Tables

**Table 1:** Nanoparticle (NP) formulations for photosensitizer (PS).

Vehicle	PS Encapsulated	Dose	Mechanism	Remarks	Ref.
Manganese ferrite MS NP	Ce6	8 mM 200 μL (i.v)	Single photon (<1 cm)	Dramatically inhibited tumor growth	[45]
Poly(d,l-lactic-co-glycolic acid) (PLGA)	MB	10 mg/kg (i.v)	Single photon (<1 cm)	Complete response in NP with PDT group	[44]
Perfluorocarbon	IR780	7.8 μg IR780 (i.t)	Single photon (<1 cm)	Inhibited 80% of tumor growth	[68]
Manganese dioxide NP	Indocyanine green	3.6 mg/mL (i.v)	Single photon (<1 cm)	Complete response in NP with PDT group	[69]
NaYF_4_:Yb,Tm	TiO_2_	0.1 g/tumor (i.t)	Single photon ^a^ (1–2 cm)	50% of the animals surviving up to 45 and 55 days	[70]
NaYF_4_:Yb^3+^, Er^3+^	graphene quantum dot		Single photon ^a^ (1–2 cm)	Tumor inhibition efficacy ~70.2%	[71]
NaYF_4_	Ce6	32 mg/kg (i.t)	Single photon ^a^ (1–2 cm)	Tumors on 70% mice disappeared in two weeks	[72]
NaYF_4_:Yb,Tm @SiO_2_	TiO_2_	0.1 g/tumor (i.t)	Single photon ^a^ (1–2 cm)	Inhibited 87.5% of tumor growth	[73]
MS NP	PS22	16 mg/kg (i.v.)	Two photon (≥2 cm) ^b^	Inhibited 71% of tumor growth	[49]
MS-Encased Au NR	PdTPP	16 mg/kg (i.t)	Two photon (≥2 cm) ^b^	Inhibited 77% of tumor growth	[57]
Hyperbranched polymer HCP@HPE	Ce6	0.10 mmol/kg Chlorin e6 (i.v)	Two photon (≥2 cm) ^b^	87 % of tumor growth is suppressed compared to control	[74]
DSPE-PEG 2000	PT2	100 μL, 500 μg/mL (i.v)	Two photon (≥2 cm) ^b^	No apparent tumor growth was observed for 18 days	[75]
RuCD	5-Fu	25 mg/kg (i.t)	Two photon (≥2 cm) ^b^	Tumor volume decreased by 85% compared to control	[76]

^a^ Upconversion NP; ^b^ maximum depth is limited by the available average power.

**Table 2:** Depth limitless X-ray scintillators for cancer therapy.

Nanosystem	Size	PS	Attachment Strategy	X-ray Doses	Exp. Subject	Ref.
MC540-SAO:Eu@mSiO_2_	400 nm	MC540	Pore loading	0.5 Gy, 50 kV	H1299 (in vitro, iv vivo)	[77,78]
					U87MG xenograft	
CeF_3_	7–11 nm	VP	Physical loading	6 Gy, 8 keV, 30 keV, 6 MeV	Panc1 (in vitro)	[79]
(*n*-Bu_4_N)_2_[Mo_6_I_8_(OOCC_10_H_15_)_6_]	50 nm	self	Encapsulated	100 keV	N/A	[60]
LaF_3_:Tb	25–44 nm	RB	Pore loading	75 kV, 20 mA	N/A	[80]
	50–150 nm	RB	Covalent binding	75 kV, 20 mA	N/A	[65]
	15 nm	MTCP	Physical loading	13.2 Gy, 250 keV	N/A	[81]
LaF_3_:Ce	2 μm	PPIX	Physical loading	2 Gy, 90 kV, 5 mA	PC-3 (in vitro)	[82]
ZnO/SiO_2_	80–100 nm	ZnO	Coating	2-10 Gy, 200 kVp, 20 mA	LNCaP and Du145 (in vitro)	[63]
GdEuC12 micelle	4.6 nm	Hyp	Physical loading	400 mA	Hela (in vitro)	[83]
N/A		PPIX	Covalent binding	8 Gy	PC-3 (in vitro)	[84]
LiYF_4_:Ce@SiO_2_	50 nm	ZnO	Coating	8 Gy, 220 keV	HeLa xenograft	[85]
TiO_2_-Tf-Tc	108 nm	TiO_2_	N/A	Cerenkov radiation	HT1080 xenograft	[33]
Cu-Cy	50–100 nm	self		5 Gy	MCF-7 xenograft	[62]
ZnS:Cu,Co	4 nm	TBrRh123	Covalent binding	2 Gy, 120 kVp	PC-3 (in vitro)	[86]
Tb_2_O_3_	10 nm	porphorin	Covalent binding	44 kV, 40 mA, 5.4 mGy/s	N/A	[66]
Y_2_O_3_	12 nm	PS	Covalent binding	2 Gy, 160 or 320 kVp	PC-3 (in vitro)	[32]
Gd_2_O_2_S:Tb	20 μm	Photo II	Colocation	130 kVp, 20 mA	Human glioblastoma	[87]
SiC/SiOx nanowires	20 nm	H2TCPP	Covalent binding	2 Gy, 6 MV	A549 (in vitro)	[88]
AuNPs	12 nm	verteporfin	Covalent binding	6 Gy, 6 MV	Panc 1 (in vitro)	[64]
CdSe@ZnS	2.1 nm	N/A	N/A	100–600 cGy/min, 6 MV	H460 (in vitro)	[67]
